# The Role of Sodium Hydrogen Exchanger 1 in Dysregulation of Proton Dynamics and Reprogramming of Cancer Metabolism as a Sequela

**DOI:** 10.3390/ijms20153694

**Published:** 2019-07-28

**Authors:** Rosa Angela Cardone, Khalid Omer Alfarouk, Robert L. Elliott, Saad Saeed Alqahtani, Samrein B. M. Ahmed, Ahmed N. Aljarbou, Maria Raffaella Greco, Stefania Cannone, Stephan Joel Reshkin

**Affiliations:** 1 Department of Bioscience, Biotechnology, and Biopharmaceutics, University of Bari, 90126 Bari, Italy; 2 Alfarouk Biomedical Research LLC, Tampa, FL 33617, USA; 3 Hala Alfarouk Cancer Center, Khartoum 11112, Sudan; 4 American Biosciences Inc., New York, NY 10913, USA; 5 The Sallie A. Burdine Breast Foundation, Baton Rouge, LA 70806, USA; 6 The Elliott-Elliott-Baucom Breast Cancer Research and Treatment Center, Baton Rouge, LA 70806, USA; 7 Clinical Pharmacy Department, College of Pharmacy, Jazan University, Jazan 45142, Saudi Arabia; 8 College of Medicine, University of Sharjah, Sharjah, UAE; 9 College of Pharmacy, Qassim University, Buraydah 51452, Saudi Arabia

**Keywords:** pH and cancer, pH and Warburg metabolism, proton transport in cancer, Na^+^/H^+^ exchanger, tumor metabolic microenvironment

## Abstract

Cancer cells have an unusual regulation of hydrogen ion dynamics that are driven by poor vascularity perfusion, regional hypoxia, and increased glycolysis. All these forces synergize/orchestrate together to create extracellular acidity and intracellular alkalinity. Precisely, they lead to extracellular pH (pH_e_) values as low as 6.2 and intracellular pH values as high as 8. This unique pH gradient (∆pH_i_ to ∆pH_e_) across the cell membrane increases as the tumor progresses, and is markedly displaced from the electrochemical equilibrium of protons. These unusual pH dynamics influence cancer cell biology, including proliferation, metastasis, and metabolic adaptation. Warburg metabolism with increased glycolysis, even in the presence of Oxygen with the subsequent reduction in Krebs’ cycle, is a common feature of most cancers. This metabolic reprogramming confers evolutionary advantages to cancer cells by enhancing their resistance to hypoxia, to chemotherapy or radiotherapy, allowing rapid production of biological building blocks that support cellular proliferation, and shielding against damaging mitochondrial free radicals. In this article, we highlight the interconnected roles of dysregulated pH dynamics in cancer initiation, progression, adaptation, and in determining the programming and re-programming of tumor cell metabolism.

## 1. Introduction

Malignant transformation of a normal cell is the first step in the evolutionary arc of cancer [1]. While the role of oncogenes and tumor suppressors in this ‘passage’ is well known, the role of ion transporters is less clear, although it is very fundamental to understand the early biological processes of tumorigenesis [2,3]. Earlier studies using the introduction of different oncoproteins into cells observed a cytoplasmic alkalinization co-occurring with a metabolic reprogramming towards glycolysis in the presence of oxygen, e.g., ‘Warburg metabolism.’ The many suggested mechanisms to explain the metabolic transformation resulting in the Warburg effect include:

(i) Adaptation to transient hypoxia;
(ii) Insulin resistance (cancer cells characterized by insulin resistance);
(iii) Abnormal enzyme content, alteration of enzymatic, and isozymatic activities;
(iv) Problems of compartmental transport translocation of pyruvate to the mitochondria;
(v) Abnormal content of mitochondria, as well as decreasing the mitochondrial number, and changing the quality of mitochondria (morphology anatomy, physiology of the mitochondria);
(vi) Abnormal electron transport and decreasing ATP production; and
(vii) Oncogenes and suppressor genes [1,4,5].

Recently, the role of intracellular pH has been gaining increasing importance as an integral and straightforward approach to explain the Warburg effect [6,7]. This work presents the current understanding of the role of pH and the NHE1 in driving transformation and determining the appearance of other ‘hallmark’ cancer characteristics [8].

## 2. pH Gradient

Both ion transport and cytoplasmic pH play critical roles in many cell functions, including management of cell growth and proliferation, growth factor kinetics, cell membrane potential, mitochondrial activity, cell volume, enzyme activity, nucleic acid, differentiation, oncogenesis, and oncogene action [9,10,11,12,13,14,15]. Much evidence over the last years has demonstrated that, clinically, all tumors have in common a critical characteristic: The aberrant regulation of hydrogen ion dynamics [9,10,11,12,13,14,15,16]. Cancer cells control the acid-base balance in a way that is entirely different from that observed in normal cells; and thereof, they produce an abnormal/extracellular acidic microenvironment, interstitial (pHe) linked to a ‘malignant’ alkaline intracellular pH (pHi). Tumor cells have alkaline pHi values of 7.12–7.7 vs. 6.99–7.05 in normal cells, while producing acidic pHe values of 6.2–6.9 vs. 7.3–7.4 in normal cells. This creates a reversed pH gradient (∆pHi/∆pHe) across the cell membrane that is markedly displaced from the electrochemical equilibrium of protons, and that increases as the tumor progresses. This specific and pathological reversal of the pH gradient in cancer cells and tissues compared to normal tissue completely alters their thermodynamic molecular energetics, regardless of their pathology and genetic origins, and can now be considered to be a defining characteristic of tumor cells [12,14,15]. Of course, the induction and/or maintenance of intracellular alkalinization and its subsequent extracellular acidosis have repeatedly been considered as playing a pivotal role in the maintenance and active progression of the neoplastic process [9,10,11,12,13,14,15].

The development and maintenance of this reversed pH gradient are directly due to the proton (H^+^) secretory ability of the tumor cells. This reversed pH gradient increases with increasing tumor aggressiveness [10,12] and local tumor hypoxia [17]. This proton production relies on the cells capacity to buffer protons and is managed by many transporters and proteins, e.g., the monocarboxylate transporters (MCT) (AKA lactate-proton symporter), vacuolar H^+^-ATPases, the H^+^/Cl^−^ symporter, carbonic anhydrases (CAs), the Na^+^-dependent Cl^−^/HCO_3_^−^ exchangers, and ATP synthase (for reviews see [9,10,11,12,13,14,15]).

The prevailing hypothesis most often considers the formation of the reversed pH gradient to be a characteristic of advanced, hypoxic tumors where the classical hypoxia-induced glycolytic metabolism is turned on. This creates high intracellular lactate and proton levels, with a consequent up-regulation of proton and lactate extruders to compensate the cytosolic acidity, such that the cytosol becomes alkalinized [18]. On the other hand, the inefficient washing out of protons [H^+^] and lactate from the interstitial space, due to disordered vascularization and chaotic blood flow, also contributes to microenvironmental acidity and the transmembrane pH gradient.

Nevertheless, when and how this cytoplasmic alkalinity of tumor cells initially occurs is a conundrum. There are data showing that the first steps of this pH gradient reversal take place at the earliest steps of malignant transformation and is tightly associated with the first observance of glycolysis in the oxygenated environment (termed the Warburg effect).

## 3. Overexpression of NHE-1 is the First Event During Malignant Transformation

This cancer cell-specific increase in proton extrusion outside the cell, resulting in the creation of proton gradient, appears during the very first steps of neoplastic transformation. Early experiments observed that ras and v-mos oncogene-dependent transformation results in a rapid cytoplasmic alkalinization, which was implicated as a crucial factor in neoplastic transformation driven by these oncogenes [19,20]. These studies also observed that these oncogene-dependent neoplastic transformations resulted in increased NHE1 activity and glycolysis, but it was not clear at the time if the driving factor for elevated pHi was the stimulated NHE1 or the increased glycolysis. This question was resolved in a study utilizing an oncogene (HPV16 E7) in an inducible vector to determine the time course of the appearance of the tumor’s hallmark characteristics, which showed that the activation of the NHE1 with the subsequent cytosolic alkalinization is the initial step in the oncogene-driven transformation of normal cells, which drove the subsequent development of a series of cancer hallmarks such as glycolysis in aerobic conditions (e.g., Warburg metabolism), increased growth rate, substrate-independent growth, growth factor independence, and tumor growth in nude mice [8,21]. By measuring the kinetic parameters of the NHE1 activity in the oncogene-transformed cells, it was found that NHE1 was constitutively activated by the oncogene expression via an increased sensitivity of its allosteric proton regulatory sites for the intracellular H^+^. This (the lowering of the H^+^-sensitivity threshold of NHE1) was responsible for its constitutive activation and the subsequent extracellular acidification with the corresponding intracellular alkalinization.

Importantly, deoxyglucose treatment decreased glycolysis to the levels of transformed cells treated with the NHE1 inhibitor, DMA, but had little effect on cellular growth in comparison with the DMA treatment [21]. Therefore, transformation-driven glycolysis appears not to play an essential role in the increase in growth rate observed upon transformation. Altogether, these data demonstrated that oncogenes utilize NHE1-induced alkalinization to produce very early the unique pH-profile and the resulting hallmark phenotypes characteristic of cancer cells [22].

## 4. The Role of NHE1 in Warburg Metabolism

A unique hallmark of cancer cells that is receiving ever-increasing attention is their metabolic reprogramming to glycolytic metabolism rather than oxidative phosphorylation (OxPHOS), even in the presence of oxygen. This was first described by Otto Warburg, and was later known as the Warburg effect [5,23]. As mentioned above, early experiments of oncogene activation displayed the first appearance of glycolytic metabolism to be an early consequence of the oncogene-induced transformation of normal cells [19,20] and were concluded by the NHE1-induced cytosolic alkalinization [21].

**Figure 1:**
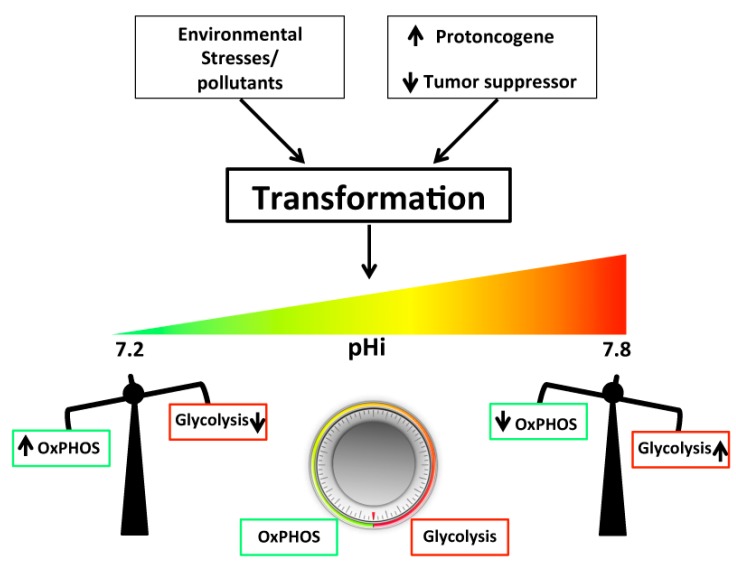
Among all the many allosteric factors controlling glycolysis, the cytosolic pH is in all probability the most significant factor regulating the metabolic balance. In the presence of adequate oxygen levels, the intracellular pH (pHi) plays a crucial role in determining the way cancer cells obtain energy: An alkaline pHi driving aerobic glycolysis, and a neutral pH driving oxidative phosphorylation with the cells pH transporters, ion channels, and enzymes, representing the core of this sophisticated and coordinated system (see text). An explanation for this phenomenon derives from the fact that both the processes of oxidative phosphorylation (OxPHOS) and glycolysis are exquisitely but oppositely pH-sensitive, and a rapid shift of metabolic cell patterns follows either acidification or alkalinization of the cytosol. On the basis of the studies discussed in this review, we can now see that the alkalinization of the cytosol occurs in the very first steps of oncogene-driven neoplastic transformation and is probably the fundamental physiological alteration utilized by the increased expression/activity of an oncogene or decreased expression/activity of a tumor suppressor to transform a normal cell. In this way, cytosolic pH would regulate the cells’ metabolic balance as a rheostat, as described in the general scheme imagined by DeBerardinis and Chandel [33], where instead of the usual on or off ‘switch’ between glycolysis and OxPHOS, they hypothesized the existence of a continuum between the two but did not have a physiological mechanism that could underlie this proposed rheostat-like mechanism.

As the cancer cell evolves, the dysregulated pHi and pHe are key regulators determining this continuing glycolytic addiction and ever-decreasing use of oxidative phosphorylation (Figure 1). Briefly, as both the processes of OxPHOS and glycolysis are exquisitely but oppositely pH sensitive, a rapid reprogramming of metabolic cell patterns follows alkalinization, probably through the modification of multiple proteins in unison to control this process. Indeed, on the one hand, alkaline pHi even slightly above steady-state levels stimulates the activity of most of the glycolytic enzymes including: Hexokinase-1 (HK-1), phosphofructokinase-1 (PFK-1), and lactate dehydrogenase (LDH), while inhibiting gluconeogenesis [5,12,24,25,26,27,28,29]. These changes in enzyme kinetics probably occur through dynamic changes in protein conformation driven by posttranslational modifications via the rapid and reversible change in the protonation of amino acid side chains (quaternary structure of the protein) [30,31]. As protonation does not require an enzyme, it supports a rapid adaptation to small shifts in pHi. The proper functioning of the mitochondrial proton transporters and proton-driven transporters that regulate OxPHOS is strongly dependent on relatively higher cytosolic proton levels [32]. Indeed, at least ten transporters regulating mitochondrial activity depend on a high, constant, regulated cytosol-mitochondrial proton gradient, including ATP synthesis by ATP synthase (Complex V), which uses energy from the proton gradient to produce ATP from adenosine diphosphate (ADP) in a phosphorylation reaction. As protonation does not require an enzyme, it permits the rapid molecules response, even to small and highly dynamic intracellular pH fluctuations. Nevertheless, the activity of several H^+^-driven transporters having a role in OxPHOS metabolism is highly dependent on the level of intracellular acidity [32].

Interestingly, there is a positive feedback mechanism where higher ATP concentrations inhibit glycolysis at phosphofructokinase 1 and pyruvate kinase 1 and, in this way, the lower ATP production resulting from a lower mitochondrial membrane proton gradient would reverse this inhibition, thus increasing the importance of the glycolytic cascade [22]. Altogether, this reciprocal rheostat-like metabolic shift of glycolytic metabolism relative to OxPHOS may well be the most sensitive cellular pHi sensor of all.

Altogether, this evidence supports the idea that the driver of the metabolic shift occurring during transformation is the alkaline shift of pHi, which becomes the ‘corner-stone’ in the altered metabolism characteristic of cancer cells. Furthermore, it has been shown that the acidic pHe has strong effects on cancer cell expression of genes involved in glycolysis [34] and other metabolic pathways [34,35,36,37]. Importantly, further changes in expression patterns of several genes, including many that regulate metabolism, occur upon inhibition of the NHE1 [38].

This pH-metabolism interaction engages a dynamic, vicious cycle from the very start: Glycolysis and proliferation are stimulated by the oncogene-driven alkalinization, which generates high energy consumption, which produces cytosolic high proton levels that stimulate proton efflux, which further alkalinizes the cell that even further reduces OxPHOS and increases glycolysis. These conditions then set the stage for the later stages of metastatic progression, thus defining the pH-centric paradigm for carcinogenesis and metastatic progression. Interestingly, the accumulation of genetic defects necessary for the clonal selection of increasingly more aggressive cells is faster than theoretically predicted [39]. The cause of this has recently been resolved with the demonstration that the tumor microenvironment (TME) contributes to tumor genetic instability, which drives the selection of aggressive cells within a tumor [12,40,41,42]. Putting together these two sets of tumor characteristics demonstrates the synergistic, positive feedback interaction of phenotype and genotype, where the initial genotypic alteration produces a phenotype, which sets the stage for additional genotypic alterations.

This regulation of cellular metabolism by the NHE1 is perhaps not surprising, considering the postulated role of ancient NHEs in the origin of chemiosmotic membrane bioenergetics [43]. According to this hypothesis, ancient NHEs are thought to have played a pivotal role in the evolution of proto-cells to produce ever less permeable membranes and ever higher levels of energy production, and harnessing by allowing cells to be independent on the environmentally provided proton gradients across a ‘leaky’ membrane for producing energy [44,45].

## 5. The Role of NHE-1 in Tumor Microenvironment (TME)

As mentioned above, this elevation of pHi of the transformed cell drives obligate tumor DNA synthesis, cell cycle progression, and both substrate-independent and serum-independent growth, resulting in a pathological and stochastic increasing in cell number as well as cellular density [12,46,47]. The increased tumor cell density decreases access to the circulation, which creates a hypoxic condition that reduces the cells’ capability to run their mitochondrial oxidative respiratory chain, and increases the need to fulfill their energy demand through glycolytic metabolism and increased glucose consumption.

Economically, glycolysis is much less efficient than oxidative metabolism in the production of ATP molecules and, importantly, each round of glycolysis produces two protons, challenging the tumor cell with an ever-increasing acid load [48,49]. Thus, pHi would rapidly decline, which could be lethal if not compensated by increased proton extrusion, which results in additional acidification of the tumor microenvironment (pHe) [50]. The alkaline shift in the pHi-dependence of the NHE1 activity observed upon transformation [21] greatly increases the acid extrusion ability of the transformed cell and alkalinize the cytosol. This may comprise the first alteration during transformation that can drive the subsequent development of the acidic microenvironment characteristic of tumors.

## 6. Other pH Regulatory Systems that Could Be Co-Driving and Maintaining the Altered pH Dynamics in the Transformed Cells and the Development of the Tumor-Specific Metabolic Microenvironment (TMM)

As stated above, there is increasing evidence that oncogenic transformation modifies the metabolic machinery of cells towards the upregulation of glycolysis, with the subsequent production of protons and lactate in the cytosol. Therefore, resulting in the overexpression and/or elevated activity of pH-regulating transporters and enzymes in the adaptive response of highly aggressive cancer cells.

## 7. Monocarboxylate Transporters (MCTs)

The monocarboxylate transporters (MCTs) are a family of proton-linked plasma membrane transporters. MCTs carry molecules that have one carboxylate group—e.g., pyruvate and lactate—and ketone bodies, together with protons, across biological membranes [2,12,13,51,52,53,54,55,56]. Monocarboxylate transporters (MCTs) are members of the SLC16 gene family and are composed of fourteen members [2,51,57]. MCT-1 is expressed on the plasma membrane of the cell [58]. Based on the endosymbiosis, mitochondria and peroxisomes are thought to have originated from bacteria—those that were endocytosed by the nucleus [59,60,61,62]. Therefore, it is no surprise that MCTs are found at both the mitochondrial and peroxisomal membranes (while peroxisomes originated evolutionarily from mitochondria) [63,64]. One of the significant evolutionary steps is that MCT-1 plays a critical role in maintaining the redox status of the cell via inter-organelle lactate shuttling within the cytoplasm of the cell [65]; e.g., NADH is regenerated from NAD^+^ in the cytoplasm by lactate. The lactate is produced from pyruvate in the peroxisome, not from glycolysis, and is translocated to the cytoplasm via MCTs [65]. The presence of this shuttle is critical to maintaining the beta-oxidation of free fatty acids.

Therefore, MCTs would extrude both lactate and protons extracellularly, which would further decrease pHe. MCTs are known to have essential roles in cancer, as studies have shown that inhibition of MCT1, both in vitro [52,66,67,68] and in vivo [69], decreased pHi and retarded tumor growth. This specificity to glycolytic tumors has suggested that its presence might be used to specifically supply therapeutic substances [52]. The upregulation of MCTs would also allow the continuous conversion of glucose to lactate since lactate is produced [12] in ever higher amounts during malignant transformation. This supports a role for MCTs in the development of the transformed and malignant phenotype. MCT1 promotes tumor progression, metastasis, and recurrence, and mediates resistance to the treatment [2,70,71,72,73], making the inhibition of MCT1 a rational approach in treating and/or managing of cancer [52,74].

## 8. Carbonic Anhydrase Enzyme (CA)

Carbonic anhydrase (CA; carbonate hydro-lyase, EC 4.2.1.1) is a zinc-containing enzyme (metalloproteinase enzyme) that catalyzes the reversible chemical reaction between carbon acid dioxide to produce bicarbonate and hydrogen ion. CA in different isoforms that might reach 15 isoforms, or even more, is localized in several cellular compartments and organelles, expressed ubiquitously in all tissues and in all the phylogenetic tree [75,76,77]. Besides, its vital role in managing the buffering capacity of the cells, CA plays a crucial role in maintaining the cellular redox state of the cell [78,79].

Carbonic anhydrase (CA) activity is important in regulating tumor cell pH [12,13,67,80], and in maintaining alkaline pHi in small tumor spheroids [68]. CAIX was recently found to be broadly localized in the interior of rat brain C6 tumor [56]. CAIX is one of the most important upregulated proteins by HIF-1alpha in response to hypoxia, both in normal and cancerous tissues [81] and is becoming important therapeutic target—e.g., SLC-0111 [12,13,54,80,81,82]. Since another consequence of the transformation/pHi-driven upregulation of glycolysis would also be the subsequent over-production of CO_2_ in the cytosol, it is possible that even in the absence of hypoxia, the transformed cell up-regulates the expression and/or activity of a CA isoform. Other pH-regulating transporters whose overexpression and increased activity play essential roles in transformation and progression are V-ATPase [12,13,83,84] and Cl^−^/HCO_3_^−^ exchangers [17].

## 9. The Role of NHE1 in Angiogenesis

The increasing hypoxia of the tumor also necessitates a new blood supply that is achieved through neoangiogenesis, whereby new blood vessels are formed from preexisting ones [69]. However, neoplastic vascularization occurs uncoordinatedly, resulting in a chaotic, functionally poor vasculature incapable of fulfilling tumoral demands of oxygen and serum and causing an inefficient washout of metabolic products (i.e., carbonic acid), which even further exacerbates the low pHe. This may be due to the unusual structure of tumor vasculature that consists of normal endothelial cells that are incorporated with malignant cells, which reflects an unusual blood vessel physiology [85].

The physiological environment, tumor metabolism, angiogenesis, and vascularization are intricately connected, which characterizes the tumor-specific metabolic microenvironment (TMM) defined by dynamic, interacting areas within tumors of:

(i) Hypoxia;
(ii) Decreasing nutrients supply (e.g., glucose and oxygen); and
(iii) Acidic pHe.

Many studies have demonstrated that both low tumor extracellular, interstitial nutrient levels, and acidity (pHe) can confer, independently of hypoxia, an evolutionary advantage for progression and eventual metastasis via substantial alterations in gene expression [86,87,88]—and has also been associated with tumor progression by impacting multiple processes, including increased invasion [14,86,89,90,91] and metastasis [14,87,92,93]. In this context, low nutrient concentrations [14,94,95,96] or low pHe [14,15,96,97,98] have been shown to preferentially stimulate NHE1 activity in tumor cells, but not in normal cells. This further drives a vicious, positive-feedback cycle, which by linking these TMM components into a dynamic, reciprocal system increasingly drives additional tumor microenvironment acidity and neoplastic progression, beginning with the first moments of malignant transformation. Importantly, NHE1 plays a critical role in integrating these interactions.

The extracellular matrix (ECM) is an extracellular macro-molecular structure (e.g., collagen, enzymes, and glycoproteins) that represents structural and biochemical support of surrounding cells. The composition of the ECM is evolved and varies greatly in tissue uniqueness functionality [99]. The ECM is constantly undergoing a remodeling process, by which components are degraded and modified, facilitated primarily by ECM proteinases [100,101]. The delicate balance between degradation and release of ECM, managed robustly by ECM-modifying cells, is responsible for tensional homeostasis and the properties of each organ, such as elasticity and compressive/tensile strength [102]. Therefore, the ECM dynamics change with changing of cellular state—e.g., during growth, division, dormancy, or even migration.

Tumor interstitial acidity strongly supports ECM dynamics during tumor progression [103]. This support acts directly through affecting the ECM dynamics or ECM-modifying cells—e.g., acidic pH increases collagen formation and assembly, which is beneficial for tumor invasion and metastases, while alkaline pH increases collagen breakdown [100,101,104,105]. Moreover, acidic pH not only affects the collagen product, but also the biology of the collagen-secreting cells [106,107].

Pavlides et al. show that the tumor stroma has a higher glycolytic rate that releases lactate, “the reverse of the Warburg effect” [1,108]. We propose—and have lately supported it experimentally—that tumor colonies act as an integrated metabolic ecosystem [1,44,109]. Cancer cells that are positioned next to blood vessels absorb the lactate from the stroma, as well as the hypoxic cells (further from a blood vessel), and consume it to complete Krebs’ cycle without glycolysis [1,66]. Therefore, pH kinetics are highly orchestrated by proton transporters. These oxidative-type cells might show higher activity of synthesis of fatty acid; as tumor cells activate de novo fatty acid synthesis to produce essential structural elements and other intermediates to produce signaling molecules. Indeed, most of the enzymes of the de novo biosynthesis pathway require conditions very similar to the cancerous one, either for cytoplasmic utilization and/or fatty acid transport [110,111].

## 10. NHE1 Inhibitors

Since NHE1 lies within the core of the carcinogenesis process, its inhibition becomes a promising approach in the treatment of cancer [10,11,112,113]. NHE1 inhibition has a direct impact on tumorigenesisa and tumor growth, inhibits metastasis, and can also defeat chemotherapy resistance [114]. Furthermore, it has been shown that NHE1 inhibition has high efficacy in the management of neuropathic pain, but not nociceptive pain [115,116,117]; thus, its effect on the latter is controversial [118].

Inhibition of NHE1 can be direct by antagonizing the activity of the ion transport or indirect via inhibition of NHE1 enhancers [119]. Several pharmacological agents act as NHE1 inhibitors, including Amiloride, Cariporide, and Phe699.

## 11. Conclusions

In conclusion, from an etiological and etiopathogenic perspective, the hydrogen-related dynamics of neoplastic transformation and malignancy have become a new approach to cancer, and their related mechanisms are helping to better understand the intimate nature of the malignant disease. This hydrogen ion-based perspective has also permitted a better understanding of the Warburg effect, which can now be explained as the result of the concerted action of proton transporters in increasing pHi and stimulating aerobic glycolysis, while reducing OxPHOS. The explanation for this phenomenon derives from the exquisite but opposite pH-sensitivity of both OxPHOS and glycolysis, and the rapid shift of metabolic cell pattern following either acidification or alkalinization. Indeed, among all the many allosteric factors controlling glycolysis, the cytosolic pH is probably the most significant factor regulating the metabolic balance (Figure 1). This unifying thermodynamic view, derived from the “pH-centric paradigm”, now permits integration of different cancer fields, ranging from cell transformation and metabolism to local growth and invasion, neovascularization, and the activation and progression of the metastatic process.

This also implies that the targeting of these transporters and ion channels would represent a new class of potential anticancer treatments and combination strategies that contribute to the war against cancer.
